# Similarity between class A and class B G-protein-coupled receptors exemplified through calcitonin gene-related peptide receptor modelling and mutagenesis studies

**DOI:** 10.1098/rsif.2012.0846

**Published:** 2013-02-06

**Authors:** Shabana Vohra, Bruck Taddese, Alex C. Conner, David R. Poyner, Debbie L. Hay, James Barwell, Philip J. Reeves, Graham J. G. Upton, Christopher A. Reynolds

**Affiliations:** 1 School of Biological Sciences, University of Essex, Wivenhoe Park, Colchester CO43SQ, UK; 2 Department of Mathematical Sciences, University of Essex, Wivenhoe Park, Colchester CO43SQ, UK; 3 Warwick Medical School, University of Warwick, Coventry CV47AL, UK; 4 School of Life and Health Sciences, Aston University, Aston Triangle, Birmingham B47ET, UK

**Keywords:** calcitonin gene-related peptide, GCR1, molecular dynamics, family B G-protein-coupled receptor, family A G-protein-coupled receptor, motifs

## Abstract

Modelling class B G-protein-coupled receptors (GPCRs) using class A GPCR structural templates is difficult due to lack of homology. The plant GPCR, GCR1, has homology to both class A and class B GPCRs. We have used this to generate a class A–class B alignment, and by incorporating maximum lagged correlation of entropy and hydrophobicity into a consensus score, we have been able to align receptor transmembrane regions. We have applied this analysis to generate active and inactive homology models of the class B calcitonin gene-related peptide (CGRP) receptor, and have supported it with site-directed mutagenesis data using 122 CGRP receptor residues and 144 published mutagenesis results on other class B GPCRs. The variation of sequence variability with structure, the analysis of polarity violations, the alignment of group-conserved residues and the mutagenesis results at 27 key positions were particularly informative in distinguishing between the proposed and plausible alternative alignments. Furthermore, we have been able to associate the key molecular features of the class B GPCR signalling machinery with their class A counterparts for the first time. These include the [K/R]KLH motif in intracellular loop 1, [I/L]xxxL and KxxK at the intracellular end of TM5 and TM6, the NPXXY/VAVLY motif on TM7 and small group-conserved residues in TM1, TM2, TM3 and TM7. The equivalent of the class A DRY motif is proposed to involve Arg^2.39^, His^2.43^ and Glu^3.46^, which makes a polar lock with T^6.37^. These alignments and models provide useful tools for understanding class B GPCR function.

## 1. Introduction

Homology modelling of class A (family A) G-protein-coupled receptors (GPCRs) is greatly facilitated by recent GPCR X-ray crystal structures [1], enabling a wealth of functional studies to be interpreted in the light of structure. For the class B GPCRs [2], including medically important targets such as secretin, glucagon-like peptide 1 (GLP-1) and calcitonin gene-related peptide (CGRP) receptors [2], modelling has been a difficult option because class A and class B GPCRs are remote homologues. Indeed, it is widely believed that class B GPCRs share virtually none of the class A conserved motifs [2–4].

Nevertheless, a number of alignments and molecular models have been developed [5–13], some based on sequence alignment [7,8,10–12] and some based on energetic criteria (in the broadest sense) [5,13,14]. Most noteworthy in this area was Frimurer & Bywater's [7] cold spot alignment of the helical regions, which was developed on the hypothesis that although the identity and properties of the residues are not conserved between the two GPCR classes, the positions of the functionally important residues are conserved.

We have developed a new approach to the difficult class A–class B alignment based on the observation that GCR1, the only well-characterized plant GPCR [15,16], has sequence similarity to both class A and class B GPCRs; its class E homologues have been proposed as the ancestral sequences for class A and class B GPCRs [17]. Thus, although standard alignment techniques fail to present a convincing alignment between class A and class B GPCRs, we can use the class A-GCR1 and the GCR1-class B alignments to define the class A–class B alignment. To support the alignment, we have further developed the principles introduced by Frimurer & Bywater [7] in their alignment and by Baldwin *et al.* [18] in the meticulous development of the once widely used rhodopsin Cα-template structure; this involved novel uses of conservation and hydrophobicity data.

In order to test the alignment against experiment, we have used data from 122 mutated residues in the transmembrane domain and loops of the calcitonin receptor-like receptor (CLR); 61 of these mutations are new. CLR is a class B GPCR that interacts with receptor activity-modifying protein 1 (RAMP1) to form the CGRP receptor: it can also interact with RAMPs 2 and 3 to give receptors for the related peptide adrenomedullin. RAMPs regulate the transport and ligand specificity of the CLR [19]. Uniquely, these mutations have been interpreted in the light of four CLR homology models generated using the same alignment but different modelling methods, namely two alternative inactive models, an active model and an active CLR–G-protein complex. The crystal structure of the active β_2_-adrenergic receptor (β_2_-AR)–G-protein complex, pdb code 3SN6, has played a key role in generating these active structures [20] and hence in interpreting the mutagenesis.

Our aim therefore was to use the novel class A–class B alignment to generate homology models of CLR so that we could interpret the wealth of experimental data on both CLR and class B GPCRs in general. The resulting active and inactive models have helped to clarify the molecular similarities between class A and class B GPCRs, and hence to increase our understanding of their activation mechanisms; this is significant because these two GPCR families are considered distinct. Moreover, while class A GPCRs are highly druggable, the development of drugs against class B GPCRs has proved difficult [21]. The availability of these alignments and structures will provide a new framework for understanding the function of class B GPCRs. Moreover, the techniques we describe of using a plant sequence and the consensus scoring to link between two distantly related membrane protein homologues may be of more widespread utility.

## 2. Material and methods

### 2.1. Profile alignment of transmembrane regions

For each helix, ungapped profile alignments between class A and class B multiple sequence alignments were scored using the Blosum 62 matrix over a sliding window of length equal to the class A transmembrane region starting eight residues before the start of the class B predicted transmembrane region and likewise continuing for eight residues after the start of the class B predicted transmembrane region. (For the purpose of the analysis, we have defined the ‘transmembrane region’ as having α-helical conformation over a range of class A X-ray structures [22], but for most of these structures, whether class A or class B, the helical region is inevitably longer by varying amounts and may contain secondary structural elements that are not canonical α-helical in varying regions.) For each of the 17 lags, the mean of the forward and reverse alignment score was taken and the score scaled over the range 0–1. The procedure was repeated for the class A-GCR1 and class B-GCR1 alignments. The highest alignment score gives an indication of the best alignment.

### 2.2. Maximum lagged correlation

This approach builds on the work of Frimurer & Bywater [7] for generating alignments that maximize the correlation of class A and class B properties, namely entropy [23] and hydrophobicity [24]. From a mathematical point of view, it is preferable to use *all* of the conservation data (as here) rather than just the data from the *most conserved* positions [7]. Consequently, we used maximum lagged correlation over the transmembrane region of the class A receptors (see the electronic supplementary material, chart S1 and text for more details). For each helix, the class A conservation data (entropy) were correlated against the corresponding class B data over a similar sliding window. For each of the lags, the mean of the forward and reverse correlation coefficients was taken and scaled over the range 0–1. The procedure was repeated for the class A-GCR1 and class B-GCR1 alignments. The highest correlation coefficient gives an indication of the best alignment.

In general, conserved residues are expected to face inwards [18] (though there will be exceptions due to GPCR oligomerization [23] and RAMP association [25]) while hydrophobic residues and residues with low conservation are expected to face outwards. Here the only condition implied by maximum lagged correlation is that the residues with similar properties tend to adopt similar positions in class A and class B receptors, and, in this respect, it is important to note that the patterns of conservation on the external lipid-facing part of the transmembrane helical bundle are similar in both class A and class B GPCRs [23].

### 2.3. Consensus scoring

For a given lag in each helix, the alignment scores, entropy correlation coefficients and hydrophobicity correlation coefficients for the class A–class B, class A-GCR1 and class B-GCR1 alignments were multiplied together to generate a consensus score. The effect of the scaling and consensus scoring was to down-weight alignments supported by just one component and to reinforce alignments that were supported by multiple components. Possible class A–class B alignments can be inferred directly or from the class A-GCR1 and class B-GCR1 alignments; peaks within 70 per cent of the maximum peak were taken as indicative of a possible alignment. For ease of reporting, the preferred alignment is reported as alignment 0 (i.e. the alignment with zero lag).

### 2.4. Variability

Variability was used by Baldwin *et al.* [18] to assist in the identification of lipid-facing regions of class A receptors. Here, we have assessed whether the patterns in transmembrane helix variability for plausible alignments are consistent with the class A structural templates over the transmembrane region.

### 2.5. Alignment probability

The probability that a given alignment could have arisen by chance was assessed by comparing the number of aligned class A group-conserved residues and class B group-conserved residues with the corresponding number in which an equivalent number of class A or class B group-conserved residues [26] were generated randomly, as described in the electronic supplementary material.

### 2.6. Numbering scheme

In the alignment determination, a negative alignment displaces the class B sequences by the stated amount to the left. This is illustrated in electronic supplementary material, chart S3, along with a description of the universal residue numbering scheme.

### 2.7. Calcitonin receptor-like receptor homology models

Four distinct homology models of human CLR were constructed using the CALRL_HUMAN sequence from Uniprot (www.uniprot.org), in line with methods published elsewhere [22,27] (the level of conservation in the CLR family range down to about 55 per cent, e.g. between human and pufferfish). These included inactive models constructed using both an implicit membrane [28] and an explicit membrane, an explicit membrane active model and a model of the CLR–G-protein complex. Implicit membrane methods have been shown to generate GPCR structures of similar quality to those generated using explicit lipid/water methods [22]. The use of different models provides an approach to understanding the uncertainty in modelling the environment of a given residue, which is most relevant in the vicinity of the loops. In addition, the use of active rather than inactive models provides a more valid reference structure for the cAMP assay experiments. Further details are given in the electronic supplementary material.

### 2.8. Calcitonin receptor-like receptor gene expression constructs and mutagenesis

The human CLR with an N-terminal haemagglutinin (HA) epitope tag (YPYDVPDYA), and human RAMP1 were provided by Dr S. M. Foord (GlaxoWellcome, Stevenage, UK) and were sub-cloned into pcDNA3.1- (Invitrogen, Renfrew, UK) prior to mutagenesis. Mutagenesis was carried out using the QuikChange site-directed mutagenesis kit (Stratagene, Cambridge, UK), as described previously [6].

### 2.9. Assay of calcitonin receptor-like receptor activation by cAMP production

CLR and RAMP1 were transiently transfected into Cos 7 cells to produce CGRP receptors as described previously. cAMP was measured by radio-receptor assay after stimulation by CGRP as previously described [6]. This measure of receptor function was chosen because of the need to test the alignment by probing as many positions as possible throughout the transmembrane region, and this measure was considered to be the most relevant to activation.

### 2.10. Radioligand binding

For a small number of mutants, the ability of human αCGRP to displace [^125^I]-iodohistidyl^8^-human αCGRP was investigated. The assays were carried out as previously described on cell membranes by microcentrifugation [6].

### 2.11. Data analysis

Curve fitting was done with GraphPad Prism 4 (GraphPad Software Inc., San Diego, USA). Both this and statistical analysis were as described previously [6].

Other aspects of the methodology are given in the electronic supplementary material.

## 3. Results

**Figure 1.**
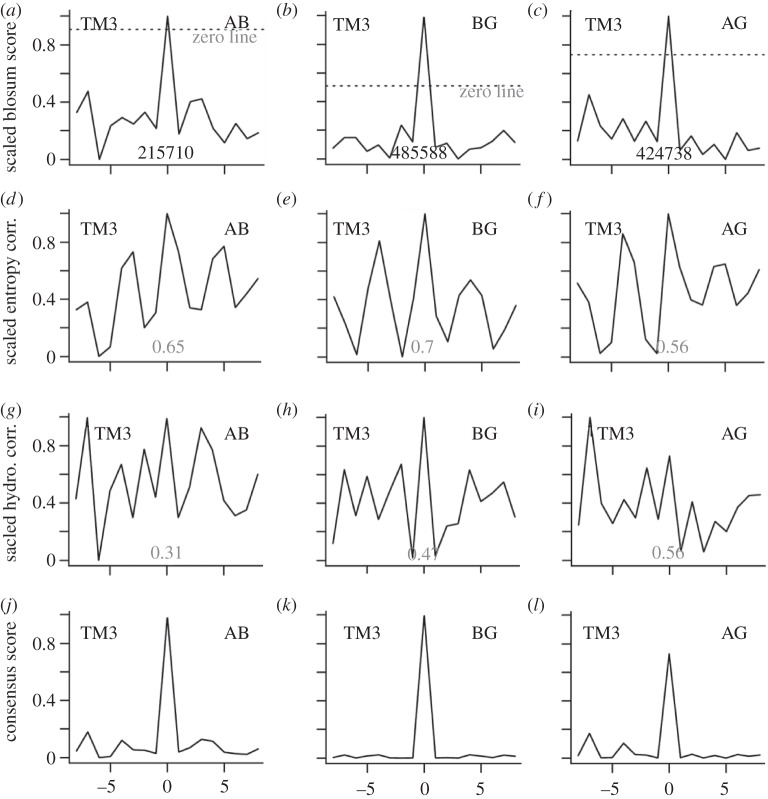
The scores for the TM3 class A–class B alignment. The top row shows the scaled Blosum 62 profile alignment scores for the class A–class B (*a*), class B-GCR1 (*b*) and class A-GCR1 (*c*) alignments; points below the dotted line have scores less than 0. The second row shows the scaled entropy correlation scores for the class A–class B (*d*), class B-GCR1 (*e*), and class A-GCR1 (*f*) alignments. The third row shows the scaled hydrophobicity correlation scores for the class A–class B (*g*), class B-GCR1 (*h*) and class A-GCR1 (*i*) alignments. The bottom row shows the consensus scores for the class A–class B (*j*), class B-GCR1 (*k*) and class A-GCR1 (*l*) alignments.

Each helix has its own characteristic degree of conservation and number of group-conserved residues [26], and its own distribution of polar and hydrophobic residues in accordance with its role in conferring stability, ligand binding, activation and G-protein coupling. Thus, for each helix, the various measures, taken in isolation, contribute differently to the identification of the preferred alignment, which can be inferred directly or preferably indirectly via GCR1. Here, our primary measure is the consensus score (figure 1*j–l*). For TM1, TM5, TM6 and TM7, the consensus score also indicates plausible alternative alignments (table 1). For this reason, additional measures have been used to distinguish between the preferred alignment and the plausible alignments, including the mutagenesis results for CLR (electronic supplementary material, table S1) and for other class B GPCRs (electronic supplementary material, table S2). Where new mutants have been made, these were initially chosen to probe the role of conserved amino acids across the TM domains. Additional mutants have been made to investigate particular regions or motifs in more detail. This approach has resulted in a good distribution of mutants across each helix.

**Table 1.** Key data relevant to alternative alignments. Data that support the preferred alignment by indicating against the alternatives shown by the consensus are shaded in grey.

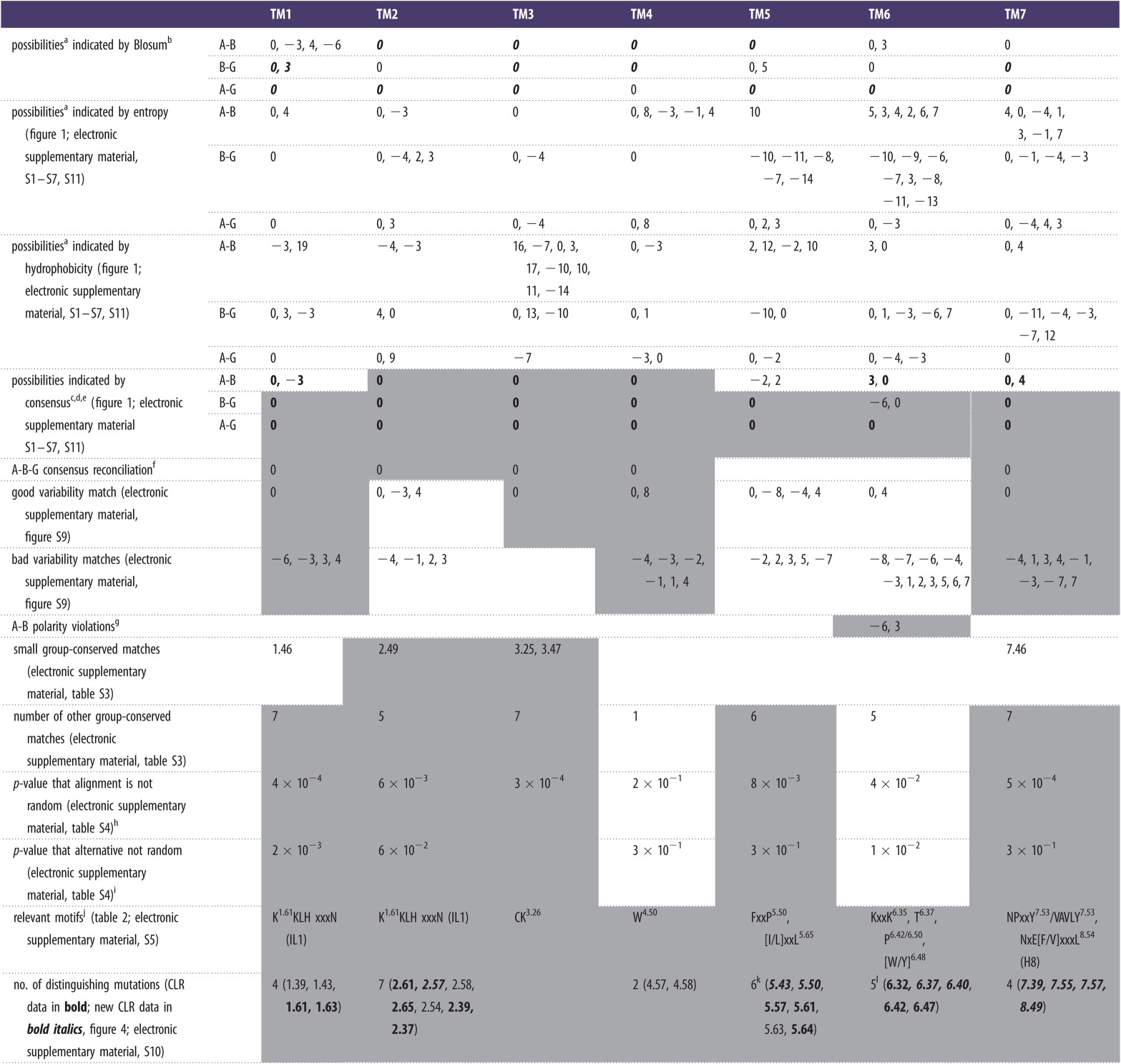

^a^Possibilities listed have scaled scores at least 80% of the maximum observed for that TM-measure category and are listed in decreasing order of size.

^b^***Bold italics*** indicates a positive Blosum 62 score.

^c^Consensus score is the product of the three separate scores, each being in the interval (0, 1).

^d^**Bold** indicates a consensus score above 0.5.

^e^Possibilities listed have consensus scores at least 70% of the maximum consensus score.

^f^Shifts that satisfy all three sets of consensus possibilities.

^g^Alignment +3 introduces 25%K/R (6.38), 3%(K/D) (6.39) and 3%R/K (6.42); Alignment −6 introduces 4%E (6.42), 3%(D/E) (6.45) and 15% (D/E) (6.50). The 0 alignment introduces 3% (R/K) (6.39). The polarity violations at 6.42 and 6.45 are severe because the charged groups cannot snorkel out of the membrane.

^h^This is based on the alignment of group-conserved residues and is the larger of the value for class A compared with random and class B compared with random.

^i^The lower of the two *p*-values for the alternative alignments is given.

^j^Because the KKLH motif (electronic supplementary material, figure S5) is part of IL1, which in turn is highly invariant in length, the motif can be considered as a continuation of both TM1 and/or TM2.

^k^For TM5, six mutations indicate against both the −2 and +2 alignments; when taken separately, eight mutations indicate against −2 and 7 against +2.

^l^For TM6, 7 mutations indicate against the −6 alignment.

In some alternative alignments, the variability pattern or the position of charged residues is incompatible with the receptor topology and this is indicated in table 1. The alignment of class A group-conserved positions with class B group-conserved positions (electronic supplementary material, table S3—this resembles the cold spot method) has been assessed by comparison with random distributions of an equivalent number of residues, and found to be statistically superior to random with a *p*-value of 0.04 or less for all transmembrane helices (electronic supplementary material, table S4, with a summary in table 1), except for TM4 where there are few group-conserved residues; the *p*-values are also generally superior to those for our plausible alternative alignments (table 1) and to those for alternative alignments in the literature. The mutagenesis data collected over 266 residues have been interpreted using the four CLR homology models. Our prime focus in *analysing* the mutagenesis data for a given residue has been to assess whether it is consistent with the alignment; seeking to understand, the function of the residue has been a secondary focus. In *reporting* the alignment, our focus has been on the motifs shared between class A and class B GPCRs, and so much of the other information is reported in the electronic supplementary material. For the majority of this mutagenesis data, it is not possible to distinguish between correct and incorrect alignments, either because the mutation had no effect, because the residue would move to an equivalent position in the plausible alternative alignment or occasionally because the homology models do not yield a consensus on the residue environment (e.g. internal versus external, helical versus loop). However, for 27 residue positions, the mutation data have been instrumental in confirming the alignment since the result is difficult to explain except in the preferred alignment; none of the mutation data are contrary to the preferred alignment. The measures in table 1 that help to define the alignment by indicating against the alternatives are shaded in grey.

### 3.1. TM3

**Figure 2.**
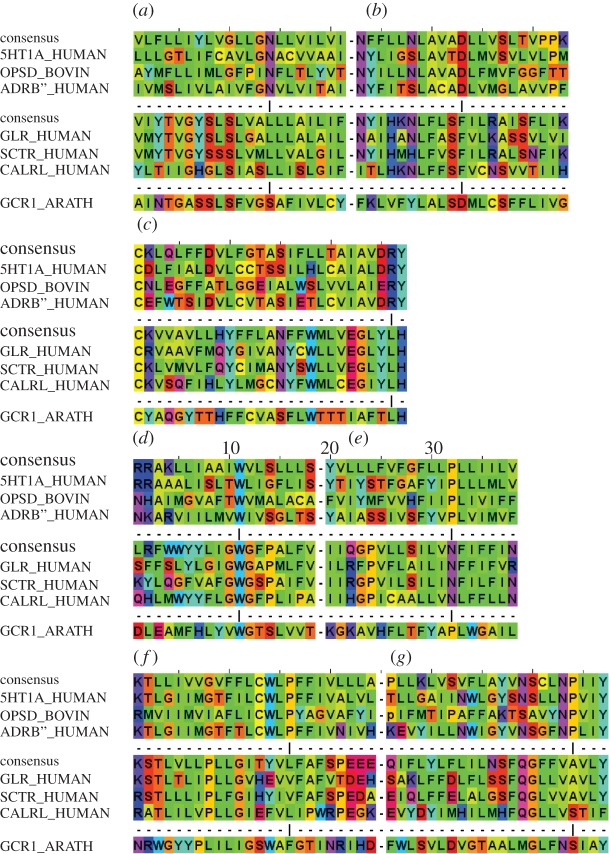
The class A–class B-GCR1 alignments (selected sequences). (*a*) TM1, (*b*) TM2, (*c*) TM3, (*d*) TM4, (*e*) TM5, (*f*) TM6 and (*g*) TM7. The most conserved positions in class A are marked by a vertical bar and correspond to position 50 in each helix, e.g. N1.50. The amino acids are coded according to their properties as follows: blue, positive; red, negative or small polar; purple, polar; cyan, polar aromatic; green, large hydrophobic; yellow, small hydrophobic. This corresponds to the ‘Taylor’ scheme, as implemented in Jalview.

The alignment for TM3 is discussed first because it is extremely clear, as shown by the consensus results in figure 1, being defined by the conserved Cys^3.26^ that forms a disulphide bond with EL2. It is also characterized by a clear positive peak in figure 1*a*–*c* for each of the three Blosum 62-based profile alignments, indicating that alignment 0 (figure 2) is the preferred alignment according to both the direct class A–class B alignment and according to the indirect method involving alignments with GCR1, as summarized in table 1, rows 2–4. Based on the entropy correlations, figure 1*d*–*f*, the class A–class B alignment gives a clear preference for alignment 0 (row 5). While not every measure supports alignment 0, when the three criteria (Blossum 62 profile alignment, entropy and hydrophobicity) are multiplied together, the weaker peaks in figure 1*a*–*i* are down-weighted, resulting in a strong prediction for the 0 alignment (figure 1*j*–*l* and table 1, rows 11–14). Since there are no other peaks, there are no plausible alternative alignments.

There are a number of highly conserved residues in TM3 (electronic supplementary material, table S3), most strikingly E^3.46^ and YLH^3.51^. The latter forms the class B positional equivalent of the class A DRY^3.51^ motif [7,29]. However, the YLH motif does not appear to have the same function as the class A DRY motif as the mutation effects are less marked [29]. The evidence from our data is that mutation has an effect on cell surface expression [30], and there are precedents for this with Y^3.51^ of the DRY motif. The YLH motif seems to be part of an extended hydrophobic network also involving the proximal part of IL2 that helps maintain the inactive receptor in a closed state, although IL2, but not Y^3.51^, also contacts Gs. E^3.46^ can interact with R173^2.39^ and H177^2.43^ in TM2 in our alignment; together they may form the functional equivalent of the DRY motif, as will be discussed below.

For the remaining helices, we will focus largely on the consensus results (rows 11–14) of table 1.

### 3.2. TM1

**Figure 3.**
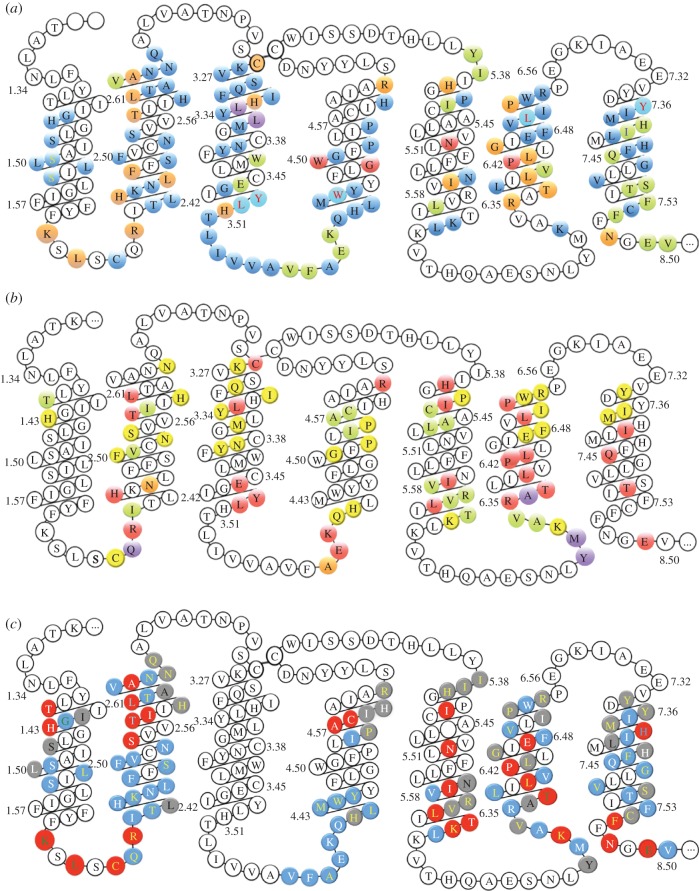
Class B GPCR mutation data (singles or doubles). (*a*) CLR mutation data. Green, orange and red shading denote a less than 10-fold, 10–100-fold and greater than 100-fold decrease, respectively, in potency for cAMP production; blue indicates no significant change. Positions that increase CGRP potency are in purple. Residues with red letters showed no change in cAMP but had decreased expression. Blue with a yellow letter indicates no information on cAMP but a change in CGRP binding. Cyan with a red letter indicates reduced expression but no change in cAMP potency. For I7.40 (green with yellow letter), there was a small increase in Kd but no change in cAMP. Further details are given in electronic supplementary material, table S1. (*b*) Mutation data on other class B GPCRs taken from the literature. Positions that show a mutation effect in CLR and in some other class B are shown in red, positions that show a mutation effect in other class B GPCRs but not in CLR are shown in yellow, positions that show an effect in other receptors but for which there is no information for CLR are shown in green and positions that show no effect are shown in orange (CLR and other receptors) or purple (other receptor; no information about CLR). Further details are given in electronic supplementary material, table S2. (*c*) Support for the alignment from the mutagenesis data. Mutation data at red residue positions help to confirm the alignment, blue residues are consistent with the alignment and grey residues are neutral. The details are as follows. Red with white lettering: functional, goes to an inappropriate position in alternative, e.g. internal/buried goes external in alternative or external residue involved in dimerization goes internal in alternative alignment. Red with yellow lettering: a G-protein contact; does not contact G-protein in alternative. Red with green lettering: key proposed interactions lost in alternative alignment. Red with cyan lettering: more prominent in binding site than in alternative. Blue with white lettering: functional goes to a similar environment in alternative. Blue with yellow lettering: not functional, e.g. because of external or buried position, goes to a similar environment in alternative. Blue with green lettering: not functional and internal goes to a similar environment in alternative. Grey with black lettering: non-functional goes to a more significant position in alternative, e.g. external goes internal or residue G-protein non-contact binds G-protein in alternative. Grey with white lettering: non-functional goes to a less significant position, e.g. internal/buried residue goes to external or G-protein contact loses contacts in alternative. Grey with yellow: inconclusive, e.g. external loop region goes to internal helical in alternative so explanations as are complicated, residues that go to different environments in different models and prolines of unknown function.

For TM1, the individual measures, profile alignment, entropy and hydrophobicity, suggest several alternatives, but 0 is the main alignment indicated by the consensus, with –3 arising as an additional lower scoring possibility from the direct class A–class B alignment (table 1; electronic supplementary material, figure S1; for TM2–TM7, see electronic supplementary material, figures S2–S7). However, the –3 alignment can be eliminated by many of the remaining measures (table 1). The mutagenesis data on CLR and on other class B GPCRs (figure 3) are especially relevant as they both support the 0 alignment and suggest how TM1 and IL1 can support G-protein interactions and receptor stability. Residues K^1.61^ and L^1.63^ are part of the KKLH^1.64^ motif that is shared between class A and class B (table 2; electronic supplementary material, tables S3 and S5); the first four residues in CLR are K167^1.61^SLS. Our models and class A X-ray structures show that L^1.63^ interacts with V391^8.50^, which in turn holds Y326^7.53^ in the inactive conformation (cf. the class A NPXXY motif) in its inactive conformation (electronic supplementary material, figure S8). K^1.61^ also interacts with G_β_ in our model of the CLR–G-protein complex—but could also interact with E^8.49^. Similar stabilizing interactions between IL1 and H8 are seen in most inactive GPCR X-ray crystal structures (e.g. rhodopsin pdb code 1U19, but not the CXCR4, pdb code 3OE6, as it unusually has a positive residue at position 8.49). None of these interactions are possible in the −3 alignment and these interactions probably underlie the loss of function on mutation of K^1.61^ and L^1.63^. With some exceptions, e.g. for splice variants, IL1 is highly invariant in length, and so the alignment for TM1 essentially defines the alignment for TM2 and hence the KKLH motif can be considered as a continuation of TM1 and/or TM2.

**Table 2.** Class A motifs and their class B counterparts.

region	class	motif	function
IL1	A	K^1.61^KLHxxxN	structure
	B	R^1.61^KLHxxxN	
TM2,3	A	DRY^3.51^	activation
	B	R^2.39^H^2.43^; E^3.46^	
TM3	A	C^3.25^	structure
	B	C^3.25^	
TM4	A	W^4.50^	structure
	B	W^4.50^	
TM5	A	IxxL^5.65^	G-protein interaction
	B	LXXL^5.65^	
TM6	A	CWxP^6.50^	activation
	B	P … TY^6.48^	
TM6	A	KxxK^6.35^	G-protein interaction
	B	KxxK^6.35^	
TM7	A	NPXXY^7.53^	activation
	B	VAVLY^7.53^	
H8	A	EFxxxL^8.54^	structure/restraint
	B	EVxxxL^8.54^	
TM2,3; TM7	A	R^3.50^–E^6.30^	ionic/polar lock
	B	R^2.39^–T^6.37^

### 3.3. TM2

For TM2, the consensus score strongly favours the 0 alignment (table 1; electronic supplementary material, figure S2). There are essentially no alternatives, as the next highest scoring alignment (−3) is well below the 70 per cent threshold. Strong support for the 0 alignment also comes from the alignment of group-conserved residues, the variability (electronic supplementary material, figure S9), the alignment of the KKLH^1.64^ motif and from the seven distinguishing mutations. Small group-conserved residues [26], which allow closer helical packing, align in TM2 and TM3 in the preferred alignment but not in the alternative alignment; in TM1 and TM7 small group-conserved residues also align in the alternative alignments. In addition, our alignment is consistent with a recent Cys-scan of the glucagon receptor which suggests that TM2 remains helical up to Q202^2.68^ [31].

### 3.4. TM4

For TM4, the consensus score strongly favours the 0 alignment, and indeed W^4.50^ aligns in all published class A–class B alignments. There are essentially no alternatives, as the next highest scoring alignment is well below the 70 per cent threshold (table 1; electronic supplementary material, figure S4).

### 3.5. TM5

For TM5, the indirect approach via GCR1 clearly favours the preferred 0 alignment. The alternative −2 and +2 alignments arise from the consensus for the direct class A–class B alignment (table 1; electronic supplementary material, figure S5). The most significant factor in determining the alignment arises out of the common interaction with the G-protein through the shared hydrophobic [I/L]xxL^5.65^ motif at the intracellular end of TM5 (table 2). Residues 5.61 and 5.65 contact the transducin C-terminal peptide in the opsin structures [32], but a larger range of residues contact the G-protein in the β_2_-AR 3SN6 structure. Mutations, particularly to polar amino acids, at position 5.61 and 5.65 in class A [33–36] and class B [37–39] GPCRs inhibit G-protein coupling. It is important to consider the G-protein interaction when seeking to understand the mutagenesis results for residues at the intracellular end of TM5 Here, the 3SN6 β_2_-AR–G-protein complex is a reasonable model since both CLR and the β_2_-AR couple to Gs.

In all, the mutation data for positions 5.43, 5.50, 5.57, 5.61, 5.63 and 5.64 (from CLR, CRF, GLP-1, PTH1 and secretin receptors) are consistent with the 0 alignment and not the ±2 alternatives (which arose from the direct alignment). The indirect alignment approach via GCR1, which does not yield any alternatives, is probably more successful than the direct class A–class B approach because of the greater divergence between class A and class B in TM5 (and in TM6).

### 3.6. TM6

**Figure 4.**
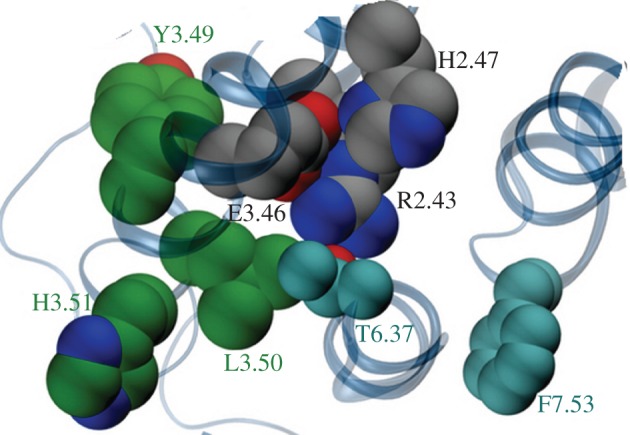
The explicit membrane inactive CLR models showing four key activation motifs in spacefill. The YLH3.51 motif is shown with green carbon atoms; the class B DRY equivalent, R2.39, H2.43 and E3.46 is shown with grey carbon atoms. F7.53 corresponding to Y7.53 of the class A NPXXY motif and T6.37, which contributes to the polar lock, are shown with cyan carbon atoms.

For TM6, 0 is the preferred alignment, with +3 as the alternative, but while all published class A–class B alignments align the conserved aromatic at position 6.48, the alignment is not as trivial as this match may imply. The class A CWLP^6.50^ motif has been much discussed as a possible activation microswitch, but since this residue does not change conformation as predicted in class A active GPCRs [20,22], its role in class B GPCRs may be equally less dramatic. Most of the mutational evidence against the +3 alignment is discussed in the electronic supplementary material, but the highly conserved T^6.37^ (electronic supplementary material, table S3) appears to be a key motif shared between class B and some class A GPCRs. This residue contacts R173^2.39^ in the inactive models but not in the active models (figure 4). Mutation of T338^6.37^ gives rise to constitutive activation in many class B GPCRs [40–43]. R173^2.39^ is part of the proposed class B DRY equivalent [7] and like T^6.37^ is highly conserved (electronic supplementary material, table S3). For these reasons, it is possible that the R173^2.39^–T338^6.37^ interaction contributes towards a class B equivalent of the class A R^3.50^–E^6.30^ ionic lock [44] (see figure 4 and discussion for further consideration of this residue). This polar lock does not form in the alternative alignments.

The TM6 alignment is partly complicated because the conserved prolines do not align. The conserved proline in class B GPCRs is at position 6.42 and like its class A counterpart at position 6.50 is likely to introduce a key bend in TM6, which is important during activation [6,45]; it also helps to define the alignment since in the 0 alignment it has the same orientation as P^6.50^ but any attempt to move the orientation of this proline in CLR results in a loss of function [6]. The KxxK^6.35^ motif may interact with the C-terminus of Gαs, aided by flexing of TM6 around P^6.42^ (class B) or P^6.50^ (class A). This interaction is born out by MD simulations, but the analogous residues in the β_2_-AR–Gs complex (PDB code 3SN6) are poorly resolved and so the interpretation should be used with care, despite the observations from mutagenesis experiments that implicate K^6.32^ and K^6.35^ in G-protein coupling in both class A [36,46–48] and class B [39,49–52] GPCRs. For this reason, it is important to note that the alignment is based on mutagenesis results throughout the transmembrane region and not in just one region, such as the G-protein coupling region.

### 3.7. TM7

For TM7, the consensus strongly favours the 0 alignment. Two motifs are apparent: the class B equivalent of the NPXXY^7.53^ motif is VAVLY^7.53^, of which the Y^7.53^ appears to be the most significant (F^7.53^ in CLR) and the NxE[F/V]xxxL^8.54^ motif on helix 8. N^7.57^ lies at the interface between TM7 and helix 8; it is conserved in most class B GPCRs (electronic supplementary material, table S3) and many class A GPCRs, but is a gap in the β_2_-AR. The models illustrate a possible stabilizing role as the highly conserved N388^7.57^ contacts R173^2.39^ of the class B DRY equivalent in both the active and inactive models, but would be unable to do so in the +4 alignment. The E394A^8.49^ mutant in the VPAC1 receptor decreases cAMP production; simulations show that this residue may interact with K^1.61^ in the inactive receptor (a similar interaction is seen between these positions in approximately 70 per cent of class A GPCR X-ray crystal structures), but it would not be possible in the alternative +4 alignment.

## 4. Discussion

The consensus for the indirect approach (via GCR1) favours alignment 0 more strongly than does the direct approach; the direct approach gives alignments that are inconsistent with experiment for TM5 and TM6, illustrating why this alignment has hitherto been difficult and highlighting the advantages of using GCR1/class E alignments. Our class A–class B alignment is not only consistent with the pharmacological phenotype of over 120 mutations in CLR and in excess of 140 additional reported class B GPCR mutations but is also superior to the plausible alternative alignments.

No single parameter can be an ideal indicator of receptor topology. For some of the mutants we have analysed (electronic supplementary material, table S1), we have only been able to measure the effect on cAMP production, rather than receptor expression or ligand binding. This limitation only applies to eight of the 27 key mutants and furthermore, the interpretation of these results does not generally rely on the precise mechanism of the mutation. For some residues, the environment in the preferred and/or alternative alignments, as shown by the four models is ambiguous; this is primarily because of uncertainties in loop modelling [53]. Previous experience has shown that the general overall picture to emerge from a homology model is likely to be correct but the details of individual residues might not be precise [13]. Although the mutagenesis has been interpreted using active state models, such models are certainly not sufficiently reliable to distinguish between different active states, e.g. those arising from partial agonists, promiscuous G-protein interactions or G-protein independent signalling. It is precisely for these reasons that we have based our analysis on multiple measures, including a sufficiently large number of mutations so that each helix has a reasonable number of distinguishing mutations, including some in the G-protein-binding region and some elsewhere.

### 4.1. Comparison with published alignments

Published class B GPCR models are split into two groups—those that use an alignment [7,10,11,54] and those that do not [5,13,14], but sometimes an alignment is not given [6,8]. Differences with Frimurer & Bywater's [7] influential cold spot method occur when there are no conserved aromatic residues to prove a strong steer, as in TM1 (alignment −4), TM2 (−4) and TM5 (+4). The cold spot method bears some similarity to our alignment of group-conserved residues, but a probability analysis is not usually carried out (cf. electronic supplementary material, table S4). Thus, although TM6 is difficult to align, there are no reported discrepancies between our alignments and the other reported TM6 alignments [7,8,10,11]. Differences with Bissantz *et al.*'s [12] clustal-based method occur in TM5, where two alternatives are given, namely (+4) [12] and (+3) [11] while Chugunov *et al.*'s hybrid method differs in TM5 (+5). Sheikh *et al.*'s method bears some resemblance to our maximum lagged correlation, which uses all of the data and hence overcomes some of the pitfalls of the cold spot method, but their alignment was only reported for TM3 and TM6. The discrepancies probably arise because in the absence of careful scaling, the score between different plausible alignments is likely to be small, and only Chugunov *et al.* have considered other plausible alternatives. None of the above, including Chugonov *et al.*'s [54] alternatives, concur with our TM5 alignment, thus making it novel.

### 4.2. Class B motifs

The mutations analysed in this study have been interpreted through CLR models, and in this respect the active model and models of the interaction with the G-protein have been particularly helpful. The CLR models have highlighted a number of possible molecular similarities between class A and class B GPCRs that are primarily associated with the class A DRY and NPXXY activation motifs and hence ultimately with the G-protein interaction; these motifs are summarized in table 2 and discussed below; the conservation data are given in electronic supplementary material, table S5. We are not aware of any similar comprehensive comparison of class A and class B motifs.

### 4.3. The interaction of calcitonin receptor-like receptor with Gs

Our alignment and modelling suggest a plausible mechanism by which CLR may interact with Gs upon activation. In family A GPCRs, movements of TMs 3 and 6 are crucial, in particular the breaking of the ionic lock between the bases of the two helices. Frimurer & Bywater [7] suggested that the class B functional equivalent of the DRY motif is R^2.39^, E^3.46^ and Y^3.49^; we concur on the role of R^2.39^ and E^3.46^. We have shown that R173^2.39^ is important for G-protein coupling [30]. Mutation of this residue in the GLP-1 receptor results in a reduction of cAMP production and mutation in the secretin receptor reduces intracellular Ca^2+^. Position 2.39 contacts the G-protein in our model complex and in the structure of the β_2_-AR–Gs complex, 3SN6. We propose that H^2.43^ is a likely partner to R^2.39^ in the activation motif because mutation of H^2.43^ (electronic supplementary material, table S2), like D^3.49^, either disrupts signalling or results in constitutive activation [50,55–57] and because H^2.43^ can hold R^2.39^ in place when E^3.46^ is mutated to alanine (simulation results not shown). The role of E^3.46^ in holding R173^2.39^ and H177^2.43^ in the inactive conformation is evident in the models. The relatively small (10-fold) effect of mutation on E233^2.46^ may arise because the reduced interaction between E233^3.46^ and R173^2.39^ and H177^2.43^ results in a receptor that has more active state character and so agonist binding is not inhibited. When the R173^2.39^–H177^2.43^–E233^3.46^ motif is considered with other key residues, the activation motif forms a more contiguous cluster than in class A GPCRs. The major changes in the active structure motifs from the configuration shown in figure 4 is that R^2.43^ drops down, the interaction with T^6.37^ is lost, while F^7.53^ moves in from its interaction with the KKLH^1.64^/EFxxxL^8.54^ cluster to help stabilize the active form of the receptor. In class B GPCRs and many class A GPCRs, there are no obvious equivalent ionic interactions between the intracellular end of TM6 and the intracellular end of TM2 or TM3 to form a canonical ionic lock. In the inactive receptor, R^2.39^ contacts T^6.37^ to form a polar lock which may hold TM3 and TM6 together. Fanelli has presented evidence that Thr (at various positions around 6.37) may also assume this role in class A GPCRs that lack a glutamate at position 6.30 [58] and indeed such an interaction is present in the inactive μ-opioid crystal structure [59]. TM5 and TM6 are the most difficult helices to align, despite the conserved aromatic in TM6, but both contain motifs, namely LxxL^5.65^ and KxxK^6.35^ that can engage the G-protein following movement of TM6, aided by the conserved prolines at positions 6.50 and 6.42. Group-conserved small residues that facilitate helix packing align on TM1, TM2, TM3 and TM7.

A further switch has been identified in the VPAC1 receptor between Q^7.45^, N^3.39^ and R^2.53^. R^2.53^ has been proposed to have a double role as a counterion for an Asp in the agonist, thus providing a switch mechanism [43]. In CLR, position 2.53 is an Asn and mutagenesis studies only indicate a role for Q^7.45^. However, our alignment indicates that this can interact with N^3.39^. It is likely that the role of this switch is receptor dependent. We are aware of other examples where mutations appear to have different effects in different GPCRs, e.g. at H^2.43^, which has a clear role in signalling in the PTH, GLP-1, secretin and VPAC1 receptors, but not in the calcitonin receptor, and R^4.64^, which affects potency in secretin, but not in PTH1 and at K^6.32^ which affects potency in VPAC2 and CRF1, but not in VPAC1 and GLP-1 (electronic supplementary material, table S2). Some of these effects depend on the residue that, e.g. K^6.32^ is mutated too. It is hardly surprising that there are differences between individual class B GPCRs, as these clearly exist among family A GPCRs as evidenced by, for example, the form and significance of the ionic lock in the structures of inactive receptors, illustrating that the key functional motifs are not immune from such effects. Work is needed to understand how GPCRs adapt similar molecular mechanisms to show distinctiveness. In this regard, the ideas generated through the alignments and models provide a framework for developing focused mechanistic hypotheses that can be tested through well-designed experiments.

### 4.4. Towards a model of the calcitonin gene-related peptide receptor; the N-terminus and RAMP1

A number of speculative low-resolution class B GPCR models have been developed that include the N-terminal domain [14,60], but there is currently insufficient evidence to allow detailed modelling. Despite this, it is possible to make a few observations about the possible orientation of the N-terminus. The presence of the RAMP in CLR-based complexes introduces useful additional constraints to drive the modelling. There are only five residues missing between the N-terminus of our model and the C-terminus of the CGRP extracellular domain X-ray structure [61]. In addition, the length of the loop connecting the RAMP of this X-ray structure to the TM helix (residues approx. 104–117) is probably too short to accommodate a model in which the CGRP peptide enters the helical bundle as shown in some low-resolution models [14,60], suggesting that the bound peptide may need to approach less vertically so as to bring the RAMP closer to the membrane. Studies by Miller indicate that this happens within a single protomer [62]. The extracellular loops play a key role in binding the peptide, and these are difficult to model [53] in the absence of experimental data, as shown by the unusual β-sheet region in EL2 of neurotensin receptor X-ray structure [63].

## 5. Conclusions

Overall, we present a plausible alignment for class B GPCRs that accommodates data from around 250 mutants from 10 class B GPCRs. While there are inevitably limitations in any molecular model, those presented here are consistent with experimental data. Residues implicated in ligand binding either face inwards or are at TM/loop boundaries and are concentrated in the upper third of the transmembrane bundle. It is possible to assign functions to highly conserved motifs and conserved residues. Using the results from this work as a basis for further experiment, it should now be possible to understand how these motifs work at the molecular level in different receptors such as the VPAC1 receptor and also how ligand binding triggers receptor activation.
